# Fer1L5, a Dysferlin Homologue Present in Vesicles and Involved in C2C12 Myoblast Fusion and Membrane Repair

**DOI:** 10.3390/biology9110386

**Published:** 2020-11-09

**Authors:** R. Usha Kalyani, K. Perinbam, P. Jeyanthi, Naif Abdullah Al-Dhabi, Mariadhas Valan Arasu, Galal Ali Esmail, Young Ock Kim, Hyungsuk Kim, Hak-Jae Kim

**Affiliations:** 1 PG and Research Department of Botany, Government Arts College for Men (Autonomous), Affiliated to Univerity of Madras, Chennai 600035, India; drusha77jc@gmail.com; 2 Sathyabama Institute of Science and Technology, Chennai 600119, India; Jeyanthiprabhu123@gmail.com; 3 Department of Botany and Microbiology, College of Science, King Saud University, P.O. Box 2455, Riyadh 11451, Saudi Arabia; naldhabi@ksu.edu.sa (N.A.A.-D.); mvalanarasu@ksu.edu.sa (M.V.A.); gesmail@ksu.edu.sa (G.A.E.); 4 Department of Clinical Pharmacology, College of Medicine, Soonchunhyang University, Cheonan 31538, Korea; kyo9128abcd@gmail.com; 5 Department of Rehabilitation Medicine of Korean Medicine, College of Korean Medicine, Kyung Hee University, Seoul 02447, Korea; kim0874@hanmail.net

**Keywords:** Fer1L5, dysferlin, myoferlin, vesicles, myoblast fusion, membrane repair

## Abstract

**Simple Summary:**

Fer1L5 is a dysferlin and myoferlin homologue and has been implicated in muscle membrane fusion events; myoblast fusion and membrane repair respectively during C2C12 skeletal muscle development. The role of Fer1L5 was analyzed by immunoblot analysis, biochemical fractionation, confocal microscopy and electroporation method. We demonstrated that Fer1L5 is present in low density vesicles and resistant to non-ionic detergent and shows overlapping properties with dysferlin and myoferlin. The expression of Fer1L5 was highly observed at the fusing myoblasts membranes and its expression level is gradually increase at the early stages multinucleated myotube formation. Fusion defects were observed in the Fer1L5 deficient C2C12 cells. Fer1L5 shows impaired membrane repair. Our data provide evidence that Fer1L5 is involved in aligning the adjacent myotubes close to each other for membrane—membrane fusion to increase the muscle mass for contraction during muscle development. Our data for Fer1L5 will be of great importance in the dysferlinopathy research in near future.

**Abstract:**

Fer1L5 is a dysferlin and myoferlin related protein, which has been predicted to have a role in vesicle trafficking and muscle membrane fusion events. Mutations in dysferlin and otoferlin genes cause heredity diseases: muscular dystrophy and deafness in humans, respectively. Dysferlin is implicated in membrane repair. Myoferlin has a role in myogenesis. In this study, we investigated the role of the Fer1L5 protein during myoblast fusion and membrane repair. To study the functions of Fer1L5 we used confocal microscopy, biochemical fractionation, Western blot analysis and multiphoton laser wounding assay. By immunolabelling, Fer1L5 was detected in vesicular structures. By biochemical fractionation Fer1L5 was observed in low density vesicles. Our studies show that the membranes of Fer1L5 vesicles are non-resistant to non-ionic detergent. Partial co-staining of Fer1L5 with other two ferlin vesicles, respectively, was observed. Fer1L5 expression was highly detected at the fusion sites of two apposed C2C12 myoblast membranes and its expression level gradually increased at D2 and reached a maximum at day 4 before decreasing during further differentiation. Our studies showed that Fer1L5 has fusion defects during myoblast fusion and impaired membrane repair when the C2C12 cultures were incubated with inhibitory Fer1L5 antibodies. In C2C12 cells Fer1L5 vesicles are involved in two stages, the fusion of myoblasts and the formation of large myotubes. Fer1L5 also plays a role in membrane repair.

## 1. Introduction

The mammalian ferlins are evolutionary conserved vesicle fusion proteins composed of at least 4-7tandem C2 domains and a C-terminal single pass transmembrane anchor which are implicated in membrane fusion events [1]. The ferlins show sequence and structural homology to the *C-elegans* FER-1 and synaptotagmins vesicle fusion proteins, which have been demonstrated to be involved in membrane fusion events [2,3]. The ferlin family includes: dysferlin, otoferlin, myoferlin (Fer1L1-3) together called type-I dysferlin-like and Fer1L4-6 known as type-II otoferlin-like ferlins (Figure S1A) [1,4]. Additional conserved domains DysF, Fer I, Fer A and Fer B of unknown functions are also present in ferlins (Figure S1B) [5]. To date, dysferlin, otoferlin and myoferlin have been well characterized in myoblast fusion. Among six ferlins, dysferlin and otoferlin mutations cause genetic diseases in humans: muscular dystrophy and deafness, respectively [6,7]. Dysferlin is the top characterized protein of the ferlin family, and the developed mouse models recapitulate human diseases [8]. Dysferlins contain 220–250 kDa sized proteins, which are highly expressed during skeletal muscle development: dysferlin in mature myofibers and myoferlin in myoblasts, respectively [8,9,10,11]. Loss or deficiency of these proteins mainly shows defects in myoblast fusion, resulting in immature myotubes and defects in membrane repair in patient muscle and mouse models (despite their other functions) [9,10,11]. The enhanced muscular dystrophy phenotype of the dysferlin and myoferlin suggested the pathogenicity of these two proteins [8,9,11,12]. Like dysferlin and myoferlin, null C2C12 myoblasts *Drosophila* mutant Rols also showed defects in myoblast fusion, producing bi- or tri-nucleated muscle cells and a mild dystrophic phenotype [13].

In muscle, membrane fusion is essential for the resealing of the plasma membrane and also for the growth of muscle by fusing of myoblast–myoblast membranes to form multinucleate myotubes and during regeneration of mature muscle [10,12]. Although not implicated in genetic disease directly, the *mdx* (dystrophin-deficient) muscle and regenerating muscle show high expression of myoferlin [14,15]. High level expression of myoferlin is believed to promote fusion events increasing the regeneration process during repair and therefore, it is considered as a major candidate for improving defects during these processes [8,16]. Transgenic mice overexpressing myoferlin in dysferlin-null mice were shown to be adequate in recovering the repair process in control muscle but only at the membrane level and not at the functional level [16]. Increased progressive dystrophy with additional severe T-tubule defects were reported in the dysferlin/myoferlin double-null model [8]. Previous studies have suggested that only C2A domains in all ferlins bind to phospholipids in a Ca^2+^ dependent fashion and functions as a Ca^2+^ sensor in muscle membrane fusion [8,11,17]. Ferlins have been shown to interact with EHD proteins, which function in the trafficking and recycling of many signaling molecules [17,18]. Among EHDs 1–4, EHD1 is well characterized. The EHD1 protein expression is elevated during muscle development and similar to dysferlin it is residing in the T-tubule structures. Like dysferlin null muscle, the *Ehd1* null muscle also has lengthened T-tubules suggested the possible role of EHD1 as a regulator in the endocytic recycling process [17,18].

Previous studies focused on the detailed structures and functional aspects of dysferlin and myoferlin [8]. Since Fer1L5 is subgrouped with dysferlin and myoferlin, one can expect similar functions of these proteins. Hence, we investigated the role of Fer1L5, the only NPF motif remaining, in the muscle membrane fusion events and membrane repair in the C2C12 model system. Our results showed that Fer1L5 is present in vesicles and expressed highly in fusing myoblasts and 3–4 nuclei containing small myotubes. The inhibitory nature of the Fer1L5 protein in C2C12 shows impaired myoblast fusion analyzed by fusion index and additionally, defective membrane repair.

## 2. Materials and Methods 

### 2.1. Chemicals and Antibodies

The chemicals used in this study were purchased from Sigma (St. Louis, MO, USA) BDH and VWR (St. Louis, MO, USA)). The antibodies listed herein were purchased from commercial suppliers: monoclonal dysferlin from Vision Biosystems (St. Louis, MO, USA), polyclonal caveolin from Transduction Laboratories ((St. Louis, MO, USA), monoclonal cyclin D3 from Calbiochem ((St. Louis, MO, USA), monoclonal transferrin receptor and monoclonal GAPDH antibody from Abcam (St. Louis, MO, USA). The polyclonal AHNAK antibody, KIS and monoclonal myoferlin have been previously described [19]. The antibodies to anti-Fer1L5 (shRNA studies) and monoclonal Lamin A were kindly donated by Beth McNally (shRNA studies) and C.J. Hutchison, respectively. Fer1L5 antibody was generated by CovalAb (St. Louis, MO, USA) and kindly provided by Dr. Bashir, Guide (Genbank Fer1L5 sequence AY461813). For immunofluorescence analysis, affinity purified antibody and for Western blot analysis final bleed were used, respectively. Goat anti-rabbit and anti-mouse immunoglobulins conjugated to horseradish peroxidase (Jackson lmmunoresearch, West Grove, PA, USA) and Alexa 488 and Alexa 546 conjugated goat antisera were used (Molecular Probes) as secondary antibodies for immunoblotting and immunofluorescence, respectively. 

### 2.2. Cell Culture

A well-known C2C12 mouse model skeletal muscle cell line [20,21] was used to study Fer1L5 function. Therefore, we followed the common protocol as described in this cell line during C2C12 muscle differentiation [22]. Other cell lines, such as human RD (rhabdosarcoma) cell line and human dermal fibroblast cells (HDF) were cultured in growth medium containing DMEM with 10% and 20% FCS, respectively, in the presence of 1% antibiotic.

### 2.3. Immunofluorescence and Western Blotting

Cells were fixed in 4% paraformaldehyde (PFA) solution, permeabilized, and washed in blocking buffer as usual for immunofluorescence study. Cells were then blocked in appropriate primary antibodies and stained with fluorescently labeled secondary antibodies. All antibodies were diluted in blocking buffer (5% FCS in 1X PBS) and incubated 1hr at room temperature or in the dark. The excess antibody was removed by performing three half hour washes in blocking buffer. Primary antibody dilutions used were: dysferlin, neat; affinity purified myoferlin, 1:10; affinity purified Fer1L5, 1:5. Cells were mounted in Mowiol containing DAPI (4′,6-diamidino-2-phenylindole) and imaged using the confocal microscope Carl Zeiss LSM 510 META (Scientifica Ltd., Uckfield, UK) with the 40× and 63× /1.10 oil immersion objectives. Using Image J (Scientifica Ltd., Uckfield, UK) and Adobe Photoshop 6.0 softwares (Adobe Inc., San Jose, CA, USA) all montages were assembled and processed. Using RIPA (Sigma, St. Louis, MO, USA) buffer containing Complete Mini Protease Inhibitor Cocktail (Roche Molecular Biochemicals, Basel, Switzerland) extraction of whole cell protein was made from monolayer cells for immunoblotting.

Following these cells were centrifuged at 10,000× *g* at 4 °C for 10 min and the supernatant was collected for the quantification of protein by the Lowry method. SDS gel electrophoresis (5–10%) was performed and the Mini-Protean 3 gel system (BioRad Mini PROTEAN and Mini TransBlot) was used to separate the proteins. The antibodies to dysferlin, myoferlin, Fer1L5, AHNAK KIS, transferrin receptor, polyclonal caveolin, Lamin A/C and Bin 1 were used at dilutions of 1:500, 1:50, 1:50, 1:100, 1:1000, 1:100, 1:200 and 1:100, respectively. Antibodies were incubated in 2–4% milk in 1× PBS. The secondary antibodies were diluted up to 1:10,000. 

### 2.4. Confocal Microscopy

Cal Zeiss LSM 510 META upright Confocal laser scanning cooled CCD camera microscope was used at 40× and 63×/1.10 lens for imaging. Images were assembled in multitrack mode using successive and simultaneous scanning averaging the background 4–7 times at a scan speed of 7 and a resolution of 1024 × 1024. 0.9 µm thickness of Z-stacks images were collected and processed using Image J software and Photoshop 6.0. For membrane repair assay the lookup table was set to “Fire”. Data are means ± S.E.M. Statistical analysis was performed using the Student’s *t*-test. 

### 2.5. Densitometry 

The protein proportion of each band from the Western blot was estimated against the background as a control using UVI bandmap software (UVItec, Cambridge, UK). Three different blots (triplicate) were used to calculate the mean percentage of these three values.

### 2.6. Biochemical Fractionation Studies

The properties of dysferlin vesicles were analyzed from the intracellular membrane fractions generated in C2C12 cells. Three different recommended protocols were followed as described in [23,24] for other vesicle proteins. 1 × 10^7^ C2C12 cells cultured in 12 × 90 mm dishes were washed with cold PBS and homogenized in HES buffer (255 mM sucrose, 20 mM HEPES, 1 mM EDTA (Ethylenediaminetetraacetic acid) at pH 7.4) containing protease inhibitor using a hand held Dounce Homogenizer by applying 15–20 strokes. The homogenates were centrifuged at 19,000× *g* for 20 min at 4 °C and the supernatant was recentrifuged at 40,000× *g* for 20 min to obtain the low density microsomal pellet. The 40,000× *g* supernatant was further centrifuged at 180,000× *g* for 90 min to obtain the high density microsomal pellet. The pellet obtained from the 19,000× *g* centrifugation was resuspended in HES buffer containing 1.12 M sucrose and layered onto 10 mls of 1. 12 M sucrose for centrifugation at 100,000× *g* for 60 min. The white fluffy plasma membrane layer was aspirated and diluted in sucrose free HES buffer for centrifugation to obtain sucrose free plasma membrane fractions. The 100,000× *g* pellet obtained was the mitochondrial/nuclear fraction. All pellets were resuspended in HES buffer containing protease inhibitors. Equal volumes of equal fractions were analyzed by Western blotting. Secondly, we performed continuous sucrose gradient centrifugation to analyze the buoyancy of the ferlin vesicles. 1 × 10^7^ C2C12 myoblasts were homogenized in 1.5 mL of homogenization buffer (HEPES 20 mM pH 7.4, 150 mM NaCl, 5 mM MgCl_2_, 2.5 mM EGTA, 5% sucrose + protease inhibitor cocktail). The lysed cells were centrifuged at 1000× *g* for 15 min. Following centrifugation 1 mL post nuclear supernatant was loaded on top of sucrose gradient ranging from 20–40% and ultracentrifuged at 166,000× *g* in an SW41 rotor (Beckman, Brea, CA, USA) for 12 h. Fractions were pulled together carefully from the top of the gradient for immunoreacitivity. Thirdly, resistance to non-ionic detergents was determined by resuspending a postnuclear C2C12 myoblast pellet in 0.32 M sucrose, 5 mM HEPES, pH 7.4 buffer containing protease inhibitors and supplemented with 0.5% final concentration of Triton X-100 (Sigma, St. Louis, MO, USA). After incubation on ice for 30 min the mixture was loaded onto a floatation sucrose gradient over two cushions of 1 M and 1.3 M sucrose, respectively. After centrifugation at 166,000× *g* for 18 h in a SW41 rotor (Beckman) fractions prepared for immunoblot analysis.

### 2.7. siRNA and shRNA Mediated Reduction

#### 2.7.1. Generation of C2C12 Stably Expressing GAPDH siRNA

We designed primers for the 3′ UTR region of the mouse ferlin genes using the 5′—AAG GTC ATC CCA GAG CTG AAC GG—3′. G3pdh (Glyco aldehyde 3 phosphate dehydrogenase) gene (a housekeeping gene) was used to standardize the RT-PCR analysis. 

#### 2.7.2. Fer1L5 shRNA Studies and Fer1L5 Inhibitory Antibody Loading Technique

Fer1L5 shRNAs (short hairpin RNA or small hairpin RNA) plasmids were bought from Sigma-Aldrich (St. Louis, MO, USA) to the target for Fer1L5. Fer1L5 shRNA knock down C2C12 cells were generated following the standard electroporation protocol described in C2C12 cell line by Amaxa nucleofection system (www.labmakelaar.com). Fer1L5 expression was analyzed in the Fer1L5 knock down cell line by Western blot analysis. However, we could not manage to obtain the immunofluorescence data to calculate the fusion index of the Fer1L5 shRNA treated cells. Therefore, we adopted a different method to disrupt Fer1L5 function in C2C12 cells, by loading Fer1L5 inhibitory antibody (Fer1L5 Ab) by replacing nucleofection buffer supplied by amaxa and obtained the results successfully. As controls, we used preimmune serum and peptide blocked Fer1L5 serum, respectively. The same Fer1L5 loaded cultures were also used for analyzing membrane repair. Wild type C2C12 cells in the presence or absence of calcium were additionally used as controls. 

### 2.8. Membrane Repair Analysis

#### Laser Injury Method to Analyze Membrane Resealing

We performed the laser injury assays in collaboration with Isabelle Richard at Genethon on wild type C2C12 cells and C2C12 cells loaded with Fer1L5 antiserum, peptide blocked Fer1L5 antiserum and preimmune serum, respectively, as mentioned in Section 2.7.2. Williams Lostal performed the wounding assay. This method measures FM-143, the fluorescence of the membrane impermeant dye, following injury of membranes by irradiation. The multiphoton confocal microscope (Scientifica Ltd., Uckfield, UK) was used to create identical wounds. In wounded cells, an increase in the fluorescence was observed when FM-143 binds to membranes and the entry of the dye stops once the injured membrane is repaired in the presence of calcium. The rate of FM-143 fluorescence influx is a measure of membrane repair following laser wounding. In the presence of the dye FM-143 membrane wound was made using multiphoton laser irradiation and the fluorescence of this dye near the wounded site was measured at 7 s intervals over a 75 s time course following membrane injury.

## 3. Results

### 3.1. Fer1L5 Expression in Different Cell Lines 

**Figure 1:**
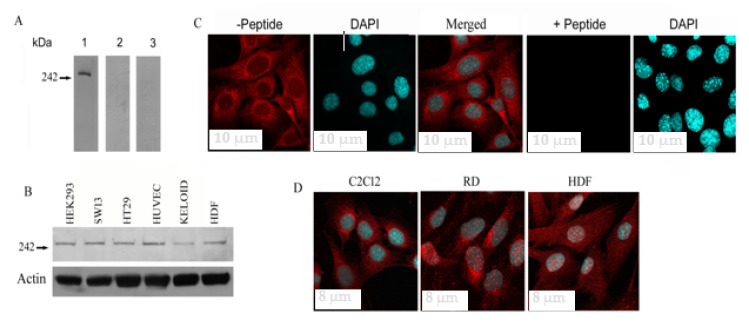
Fer1L5 protein expression in different cells. (**A**) Fe r1L5 antibody characterization. On blots Fer1L5 showed the specific band little above the predicted 242 kDa protein (indicated with arrow). Preimmune serum, blot 2; peptide blocked Fer1L5 anti-serum, blot 3. (**B**) A similar band for Fer1L5 was detected in the mammalian cell lines, HEK293 (human embryonic kidney 293 cells), SW13 (adrenal carcinoma cell line), HT29 (human colon adenocarcinoma cell line), HUVEC (human umbilical vein endothelial cells), KELOID (keloid fibroblasts) and HDF (human dermal fibroblasts) cells. (**C**) C2C12 myoblasts incubated with affinity purified Fer1L5 antibody (−peptide) and peptide blocked Fer1L5 antibody (+peptide) and the identical confocal settings were used to image the cells. DAPI stains nuclei. Scale bar, 10 μm. (**D**) Z-series sections depicting Fer1L5 expression in C2C12, human rhabdosarcoma (RD) and human dermal fibroblasts (HDF), respectively. Scale bar, 8 μm.

We began our investigation of Fer1L5 with its protein specificity in C2C12 cells. Equal amounts of protein (20 μg) prepared from C2C12 myoblast lysates were used to generate replica blots for Western blot analysis. By immunoblotting of C2C12 lysates, Fer1L5 antiserum recognized little above the predicted 242 kDa protein from uniport (AY461813.1:1-6297) which is also similar to the reported 242 kDa size predicted for Fer1L5 and the data available at https://www.mybiosource.com/polyclonal-mouse-rat-antibody/fer1l5/9140619. The size of the Fer1L5 protein is a little larger than the other two related proteins, namely, dysferlin and myoferlin and also has just, more closely to the yearly report (Figure 1A) [8,18]. Peptide blocked Fer1L5 and preimmune serum were used as controls which showed no immunoreactivity for Fer1L5. A similar band for Fer1L5 was also obtained in the different mammalian cell lines examined (Figure 1B). Fer1L5 showed a predominant punctuate staining pattern in the cytoplasm of C2C12 myoblasts signifying that Fer1L5 is present in vesicular structures in myoblasts (Figure 1C). The vesicular nature of Fer1L5 has already been reported in C2C12 cells using a different anti-Fer1L5 antibody [18] and intracellular staining for Fer1L5 in all cells analyzed with another new anti-Fer1L5 antibody [4]. Nuclear staining was also observed in C2C12 myotubes and other cells analyzed (Figure S1C). The cultured cells, such as rhabdosarcoma (RD) and HDF also displayed a similar predominant punctate staining pattern indicating that Fer1L5 is localized in vesicles (Figure 1C,D).

### 3.2. Overlapping Properties of Dysferlin Vesicles 

**Figure 2:**
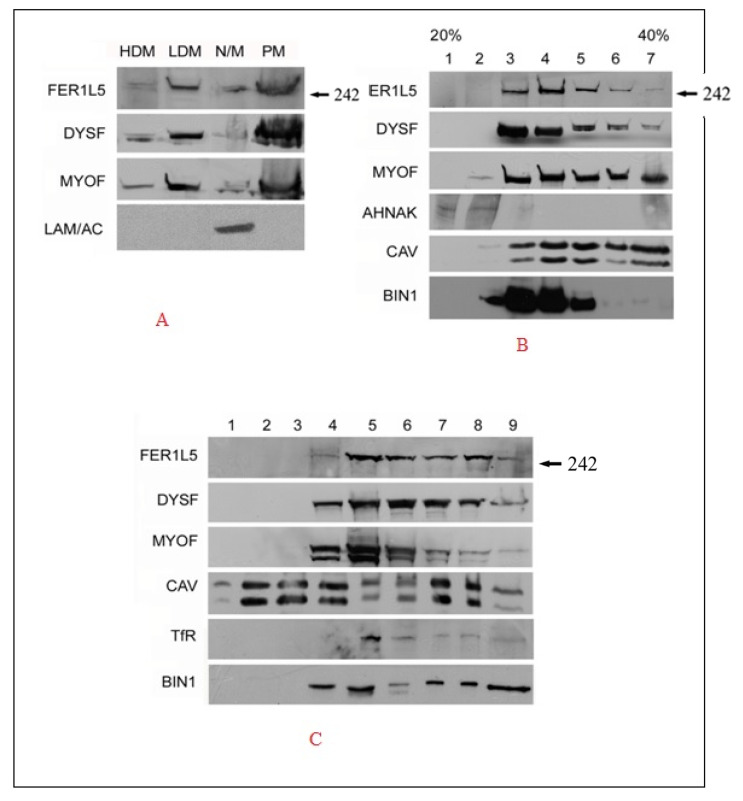
Fer1L5 Subcellular distribution in C2C12 myoblasts. (**A**) Equal volumes of each membrane fraction were analyzed. HDM = high density microsomes, LDM = low density microsomes, N/M = nuclei and mitochondria, PM = plasma membrane. Three independent trials were analyzed in total. LAM/AC is Lamin A. (**B**) A blot shows the post nuclear supernatants of homogenized C2C12 myoblasts loaded onto sucrose gradients ranging from 20–40%. Equal volumes from 7 fractions were collected for analysis. (**C**) Floated fractions 1–4; dissolved fractions 5 to 8. Fraction-9, pellet. The data presented here are representative of two independent experiments.

Our immunolabelling data suggest that Fer1L5 is present in vesicles, like dysferlin and myoferlin. Hence the subcellular distribution of Fer1L5 in C2C12 myoblast lysates was explored in more detail by biochemical fractionation studies. Equal amounts of each membrane fraction generated from C2C12 myoblasts, display a dynamic intracellular distribution of Fer1L5 being present in the vesicular, plasma membrane and mitochondria/nuclei fractions, respectively, and its subcellular distribution profile appearing most similar to other two ferlins. Lamin A was used to confirm the fidelity of the fractionation which is only enriched in the nuclear/mitochondrial fraction (Figure 2A). We then look at the relative distribution and properties of these vesicles further by fractionation on continuous sucrose density ultracentrifugation of post nuclear C2C12 myoblast supernatants. This revealed that myoferlin and dysferlin are present in both light and dense vesicular fractions (three to six). The bulk of Fer1L5 was predominantly recovered in light vesicular fractions (four and five). AHNAK, an interacting protein of myoferlin and dysferlin [25] and a marker for enlargeosomal vesicles implicated in membrane repair [23,24], was only found in the one and two lighter vesicular fractions. A muscle membrane protein, caveolin 3 (CAV-3) [26], which participates in the dysferlin endocytic pathway, shows overlapping immunoreactivity with dysferlin vesicles, respectively. BIN 1, otherwise called as amphiphysin 2 and a T-tubule marker protein [27], is involved in the trafficking of vesicles and in the generation of tubular invaginations implicated in T-tubule biogenesis [28]. In the culture myotubes BIN 1 was shown to associate with dysferlin at membrane wounding sites was co-sedimented in low and high density fractions three to five. The profile shows overlapping properties of BIN 1 with dysferlin as expected and also with myoferlin and Fer1L5 (Figure 2B). The data support the results shown for Fer1L5—that there is increased Fer1L5 staining in the malformed EHD1-null-T-tubules where it co-localizes with BIN1 [17]. We suggest that the T-tubule membrane structures are not only the source of the dysferlin vesicles, but also for Fer1L5 vesicles [10].

To obtain further evidence of the similarities of these vesicles, the resistance of the dysferlin vesicles to non-ionic detergents was demonstrated by floatation sucrose gradient centrifugation following treatment of post nuclear C2C12 pellets with 0.5% TX-100. Caveolin-3 and transferrin receptors were used as markers of detergent resistant and soluble membranes, respectively, and the results were reliable, as reported [23]. Fer1L5 shows predominant immunoreactivity in the dissolved five and eight non-floated fractions highlighting that the Fer1L5 vesicular membranes are largely non-resistant to detergent. For myoferlin and dysferlin the bulk of immunoreactivity was at the interface between the floated and dissolved fractions. The Bin 1 recovery profile closely resembled those obtained for dysferlin vesicles (Figure 2C). Overall, the profiles from co-sedimentation experiments of dysferlins vesicles overlapped with each other, highlighting their potential co-localization in C2C12 muscle cells. 

**Figure 3:**
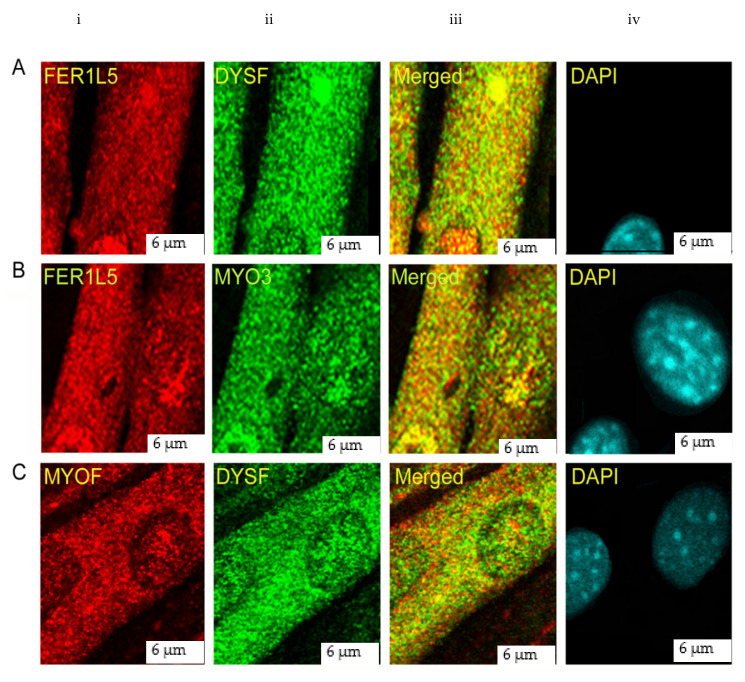
Co-localization studies. Z-series myotube sections are presented. Images showing co-localization studies. (**A**) Fer1L5 (red) channel) and dysferlin (green channel). (**B**) Red, Fer1L5; green, myoferlin. (**C**) Red, myoferlin; green, dysferlin. DAPI stains nucleus (**A**–**C**) (blue channel). Yellow colour indicates co-stained regions in the cytoplasm and nucleus (**A**–**C**), respectively. Scale bar, 6 μm.

Co-immuno labelling was performed in C2C12 myotubes because high expression of dysferlin is not observed in myoblasts. This showed Fer1L5 staining in vesicular structures and also in distinctive aggregates in all myotube nuclei which reveals partial co-localization with dysferlin in the cytoplasm (marked with a star in Figure 3Aiii) and nuclei (highlighted by arrow heads in Figure 3Aiii). In contrast to dysferlin, we observed a complete co-localization of Fer1L5 and myoferlin in the nucleus (highlighted by arrow heads in Figure 3Biii) and a partial cytoplasmic co-localization (marked with a star in Figure 3Biii). Minimal colocalization of dysferlin and myoferlin was observed in the cytoplasm (marked with a star in Figure 3Ciii) and subnuclear regions (highlighted by arrow heads in Figure 3Ciii).

### 3.3. Fer1L5 is Elevated during C2C12 Myoblast Fusion

**Figure 4:**
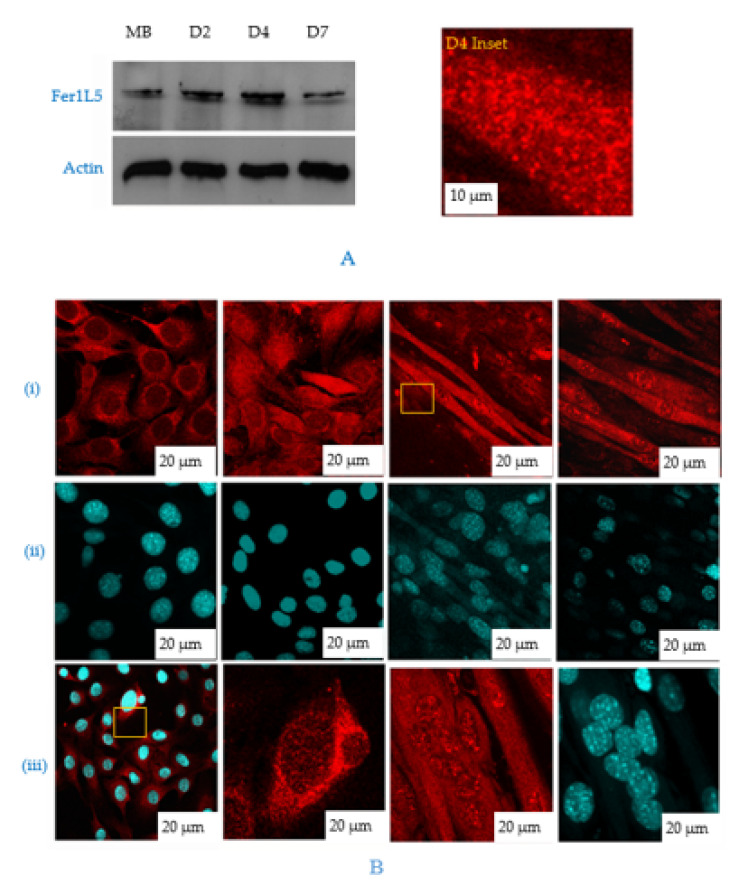
Fer1L5 expression profile during C2C12 differentiation. (**A**) C2C12 lysates collected from D0–D7. MB = myoblast, D = time in days after serum withdrawal: D2, D4 and D7. Actin was used as a control. Scale bar, 10 μm. (**B**) (**i**) C2C12 myoblast and myotube cultures at different time points. Scale bar, 10 μm (MB) and 20 μm (MT). Inset image of D4. For simplicity we presented the picture on top right. (**ii**) shows the DAPI staining in the nucleus for each time point. 0.9 μm thickness. (**iii**) Fer1L5 staining in fusing myoblasts (Scale bar, 10 μm) and myotubes (indicated by arrow head in inset image) and in the nuclei of all stages.

The overlapping properties of the dysferlin vesicles indicated that Fer1L5 has also taken part in muscle membrane fusion, as reported for dysferlin (membrane repair) and myoferlin (myoblast fusion) [11,29]. Therefore, we aimed to analyze these three ferlin’s expressions at different stages of myogenic differentiation. By Western blotting, increased expression of dysferlin was observed in mature myofibers in line with reported results from C2C12 cells and myoferlin expression was found to be higher in the mononucleated myoblasts than myotubes [8,9,10,11]. Our profile for Fer1L5 showed fluctuating expression levels during myoblast differentiation. Fer1L5 expression was highly elevated (like dysferlin) when the myoblasts are involved in maximal fusion during the early stages (D2–D4 post serum differentiation) and the expression levels were shown to be reduced moderately when the myotube matures (D7), unlike dysferlin (Figure 4A,B). This was consistent with the previous report for Fer1L5 expression at D4 [18]. By densitometry, the increase in the Fer1L5 expression levels was measured and calculated as 20% at D2 and 30% at D4. The increased expression of Fer1L5 was analyzed by immunolabeling of C2C12 cultures because these cells are non-synchronous and the differentiating cultures used to examine the overall Fer1L5 expression contained a combination of cells at various stages of myoblast fusion. In the differentiating cultures, Fer1L5 expression was slightly higher in prefusing myoblasts (D2) and the expression was increased two-fold when the cells fuse efficiently and form multinucleated myotubes (D4) and then the expression level is reduced (D7), consistent with the Western blot data (Figure 4Bi). The staining pattern for both myoblasts and myotubes was observed as described above. Fer1L5 also shows aggregate like staining in the nucleus of all myotubes analyzed. We report for the first time, the nuclear staining of dysferlin and myoferlin and this was confirmed by nuclear fractionation. The DAPI staining was used to show the nucleus in each time point (Figure 4Bii). Interestingly, we observed increased concentration for Fer1L5 in vesicular structures at membrane fusion sites of fusing myoblasts, which we did not detect in apposed membranes of myoblast–myotubes and myotube–myotubes (Figure 4Biii), as shown for dysferlin and myoferlin [10,11]. Overall, the profile for Fer1L5 is appeared to be intermediate between dysferlin and myoferlin, suggesting that Fer1L5 may operate distinctly and may be involved in the merging of apposed membranes. 

### 3.4. Inhibition of Fer1L5 Impairs Formation of Large Myotubes and Defective Membranerepair

**Figure 5:**
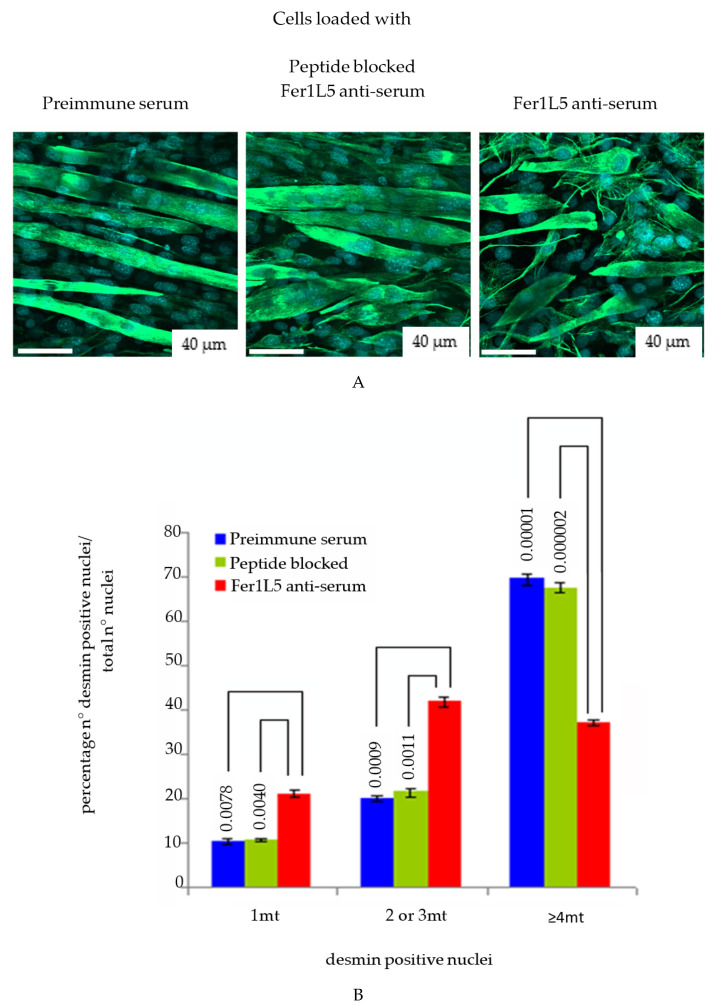
Fer1L5 inhibition impairs the formation of large myotubes. (**A**) D9 myotubes were used for fusion index. Desmin positive cells were counted in the antibody loaded myoblasts and myotubes in the 40 randomly chosen areas on the coverslips. Three independent experiments were performed in total and we counted 2792 nuclei for preimmune serum loaded cells, 2787 for Fer1L5 antiserum loaded cells and 2784 for peptide blocked Fer1L5 antiserum loaded cells. Desmin positive nuclei were identified using DAPI staining. (**B**) Histogram showing the fusion index and the *p*-values of the above three cultures.

Since there was accumulating evidence that Fer1L5 may function in C2C12 myoblast fusion we preceded knockdown studies for Fer1L5 using shRNA. ShRNAs are frequently used in functional gene studies and in the validation of novel drug targets. G3PDH siRNAs were used to optimize the electroporation protocol for C2C12 myoblasts and showed reduced expression (Figure S2A). Fer1L5 shRNA showed a considerable reduction in the Fer1L5 protein level (50–60%) by Western blot analysis (Figure S2A). Unfortunately, we did not manage to obtain immunoflurescence data, as was the case with other groups, for Fer1L5 knockdown cells [18] to calculate the myogenic index of the number of myotubes. We therefore adopted an alternative approach of antibody inhibition by Amaxa nucleofection system for Fer1L5 antibody loading, which has been used successfully to disrupt protein function and showed 40–50% knockdown in the Fer1L5 loaded cultures (Figure S2B). The fusion index of the Fer1L5 inhibitory antibody loaded cultures were determined using desmin positive myogenic cells, as described for myoferlin [11]. No changes were observed in the proliferation rate between the three groups of cultures. Upon examination Fer1L5 anti-serum displayed a considerable increase in the number of two to three nuclei containing desmin positive myoblasts and myotubes and significantly fewer large multinucleated myotubes (≥4 nuclei) (Figure 5A) consistent with the data shown for Fer1L5 siRNA transfected C2C12 cultures [18]. The fusion index of Fer1L5 antiserum loaded cultures was shown to be 37.2% which was significantly lower than that obtained for preimmune serum loaded (69.6%; P = 1 × 10^−5^) and peptide blocked Fer1L5 antiserum loaded (67.4%; P = 2 × 10^−6^) cultures, respectively (Figure 5B). Our results indicated that the formation of large myoubes (≥4 nuclei) was impaired in the presence of the Fer1L5 inhibitory antibody [18].

The above results allowed us to proceed with membrane repair in the Fer1L5 loaded cultures using the most common and accurate multiphoton laser wounding membrane repair assay, as reported for dysferlin [17,19]. The rate of FM-143 fluorescence influx is a measure of membrane repair following injury in the three different loaded cultures and wild type C2C12 cells, respectively.

**Figure 6:**
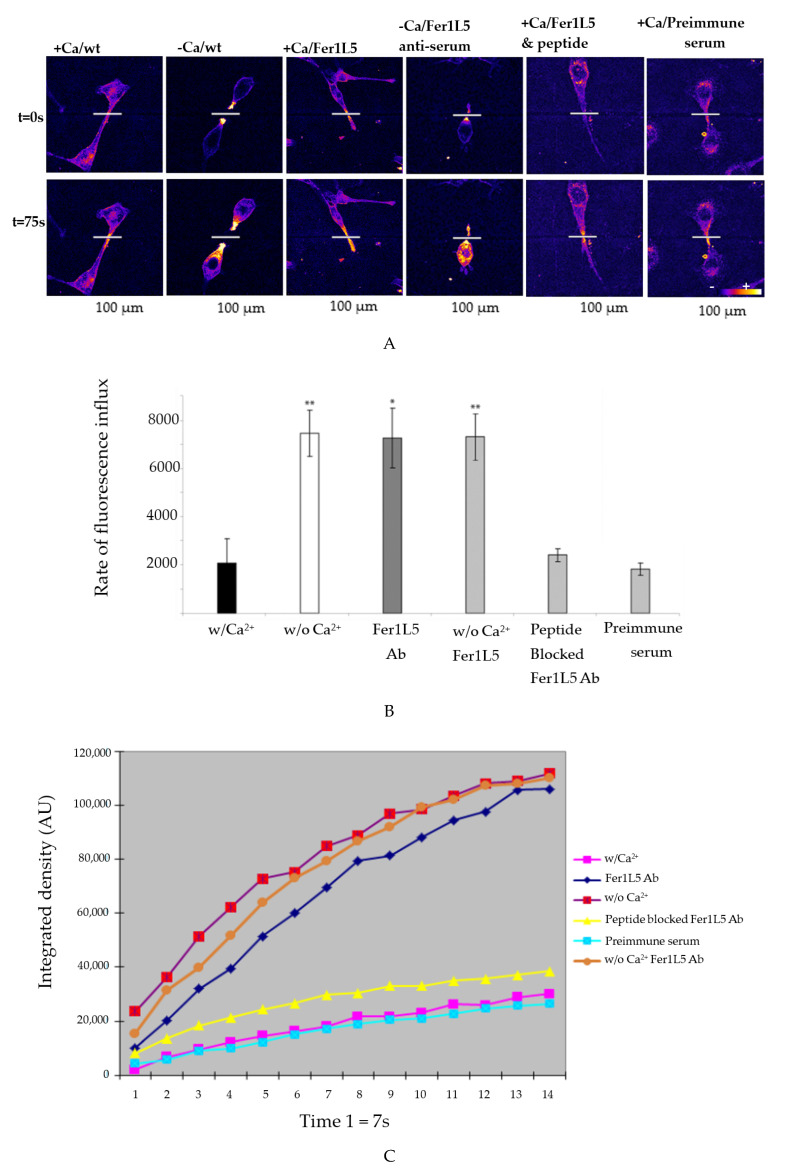
Membrane repair defects in C2C12 myoblasts loaded with Fer1L5 inhibitory antiserum. (**A**) Injury sites in the myoblasts were indicated by white lines. Time is shown in seconds. Scale bar = 100 µm. (**B**) Histogram showing the deduced rates of fluorescence influx (Δ (fluorescence)/Δt): w/(*n* = 3); w/o (*n* = 4); Fer1L5 antiserum (*n* = 4), w/o Ca^2+^ Fer1L5 antibody (*n* = 4), peptide blocked Fer1L5 antiserum (*n* = 5) and preimmune serum (*n* = 3). * indicate a *p*-value < 0.05; ** *p*-value < 0.01. (w/): with calcium. (w/o): without calcium. (**C**) Shows the rate of fluorescence influx every 7 s time duration in the wounded cells with and without Ca^2+^ and Fer1L5 antibody as indicated. Controls: Preimmmune serum, peptide blocked Fer1L5 antibody (Ab) and w/calcium.

The wound was made in the wild type C2C12 cells containing Ca^2+^ and in the C2C12 cells loaded with preimmune serum and peptide blocked Fer1L5 antiserum, respectively, showed quick patch of their torn membranes over the 75 s time course (Figure 6A). Fer1L5 antiserum loaded C2C12 cells lose the ability to hinder FM-143 dye entry during the same time course (75 s) and showed resealing defects (Figure 6A,B). Similar membrane repair defects were observed in the lack of Ca^2+^ in the wild type C2C12 cells (Figure 6B,C). These preliminary results showed that Fer1L5, like dysferlin and myoferlin, may take part in muscle membrane repair.

## 4. Discussion

In this report, we have demonstrated that Fe1L5 is involved not only in myoblast fusion, but also its role extends to membrane repair, similar to dysferlin and myoferlin [11,12,29]. By immunolabelling, the Fer1L5 antibody predominantly showed cytoplasmic vesicle staining in all cells analyzed. The vesicular nature of Fer1L5 was confirmed by biochemical fractionation and co-immunolabelling studies, which displayed that these dysferlins are present in distinct vesicles in myotubes and co-localize with each other, respectively. Overall, our results revealed that the dysf-ferlins share overlapping properties with each other and also with other vesicle and membrane proteins, suggesting that they also have overlapping functions in muscle cells, as predicted [27]. The membranes of the Fer1L5 vesicles partially overlapped with caveolin-3 and T-tubule protein, BIN1 [27], consistent with a role of Fer1L5 in muscle membrane fusion. The T-tubule membrane system functions in excitation–contraction coupling and is also recognized as an essential membrane source of vesicles [30]. The proposed roles of BIN1 are vesicle trafficking and generation of the T-tubule invaginations involved in T-tubule biogenesis [27,28]. Based on the co-localization studies, we suggest that the T-tubule membrane structures are not only the source of dysferlin vesicles, but also for Fer1L5 vesicles, which participate in myoblast fusion and membrane repair [10,17].

We suggest the lack of dysferlins and caveolin-3 or BIN1 leads to accumulation of vesicles and unusual T-tubule structures within muscle [10,31]. A model was proposed, highlighting that the above mentioned proteins along with other interacting proteins during maturation in the T-tubule and at the muscle membrane is responsible for cytoplasmic rearrangements and fusion of vesicle membranes [17]. The data are reliable for the function of regulatory proteins, the EHD protein family, in the intracellular trafficking in the endocytic recycling pathway similar to the Rab family of GTPases [32]. Because skeletal muscle, as a secretory organ, is largely reliant on this intracellular trafficking to build its unique structure and for development, it is therefore possible to suggest that the dysf-muscle ferlins are implicated in the trafficking process during muscle development and growth [4,32]. 

Next, we show that Fer1L5 is involved in myoblast fusion, as expected. In muscles, membrane fusion is essential for the growth of muscle by fusing of myoblast–myoblast membranes to form multinucleate myotubes and for the plasma membrane repair and also for the regeneration of mature muscle. Following Fer1L5 inhibition, impaired formation of multinucleated myotubes was observed (>4) similar to dysferlin null and myoferlin null myoblasts as expected [10,11,18]. However, unlike dysferlin and myoferlin vesicles, which accumulate at fusing C2C12 myoblast/myotube membranes [10,11], we observed no such accumulation of Fer1L5 at these sites, indicating that Fer1L5 may operate differently to dysferlin and myoferlin during myotube growth. These data suggest that Fer1L5 may be an early myogenic marker as dysferlin has already been shown to interact with myogenic marker myogenin. The strong Fer1L5 staining was not observed in control myoblasts, suggesting that Fer1L5 may function in the merging of apposed myoblast–myoblast membranes. In myoblast fusion, Fer1L5 is predicted to function upstream from dysferlin and myoferlin in membrane merging. Consistent with this view, Fer1L5 antiserum loaded C2C12 cultures contained a significantly increased number of myoblasts positive for desmin staining, compared to control cultures indicating that the myoblasts were primed for fusion but unable to fuse. 

Finally, we examined the membrane repair ability of Fer1L5 in the Fer1L5 inhibitory antibody loaded cultures and showed defective membrane repair as demonstrated for dysferlin and myoferlin vesicles [33,34,35]. Dysferlin is considered as sarcolemmal repair vesicles and emergency fusogen along with its binding partners involved in patching the membrane, but the origin and the identity of these vesicles is still an open question [8,12,17,25]. Studies have demonstrated that the recovery of the repair ability in the muscle of patients having dysferlin deficiency when the muscles were treated with acid sphingomyelinase (ASM) and suggested that the ASM could also be a potential therapy for dysferlinopathy [8]. Similar results were shown for myoferlin, suggesting that myoblast fusion is crucial for embryogenesis, membrane repair and muscle regeneration [8,11]. Myoferlin-null mice showed similar delayed muscle repair as dysferlin after injury, highlighting that myoblast fusion is not only required during embryogenesis but also during damage repair and muscle regeneration [8,11]. From our preliminary studies we also provide evidence that the membrane repair was disrupted in the deficiency of Fer1L5. Given the molecular similarities of Fer1L5 with dysferlin we argue that Fer1L5 may also have a role in the dysferlin repair pathway but further study will give an insight how Fer1L5 functions as a therapeutic target for the dysferlinopathies.

## 5. Conclusions

Overall, the data provided evidence that dysferlin proteins are present predominantly in vesicles and some at the plasma membrane and express at different stages of C2C12 myoblast fusion. The similar and distinct functions of Fer1L5 compared to another two ferlins: dysferlin and myoferlin during C2C12 muscle development, was highlighted in our study and showed that these three ferlins are located in the specific subcellular regions which facilitate vesicle fusion events and mediate myoblast fusion and this is depicted in our model (Figure S3A,B). Fer1L5 is also involved in membrane repair.

## Supplementary Materials

The following are available online at https://www.mdpi.com/2079-7737/9/11/386/s1, Figure S1: Schematic showing the structural similarities and subgrouping of the human ferlin protein family, Figure S2: Transfection in the Fer1L5 shRNA and Fer1L5 antibody loaded cultures, Figure S3: Fer1L5 differentiation role in myoblast.

## Abbreviations

DysF	Dysferlin-like-Ferlin
EHD proteins	Eps15 Homology Domain
DMEM	Dulbeccos modified eagles medium
BIN 1	Bridging integrator 1
MG53	Mitsugumin 53 (tripartite motif, TRIM72)
