# Supplementary material for: Fer1L5, a Dysferlin Homologue Present in Vesicles and Involved in C2C12 Myoblast Fusion and Membrane Repair
Source: Biology (Basel). 2020 Nov 9;9(11):386. doi: 10.3390/biology9110386 (PMC7695329; doi:10.3390/biology9110386)
Supplement: Supplementary file 1 [file biology-09-00386-s001.pdf]

biology

MDPI

Article

Fer1L5, a Dysferlin Homologue Present in Vesicles and Involved in C2C12 Myoblast Fusion and Membrane Repair

R. Usha Kalyani, K. Perinbam, P. Jeyanthi, Naif Abdullah Al-Dhabi, Mariadhas Valan Arasu, Galal Ali Esmail, Young Ock Kim , Hyungsuk Kim and Hak-Jae Kim

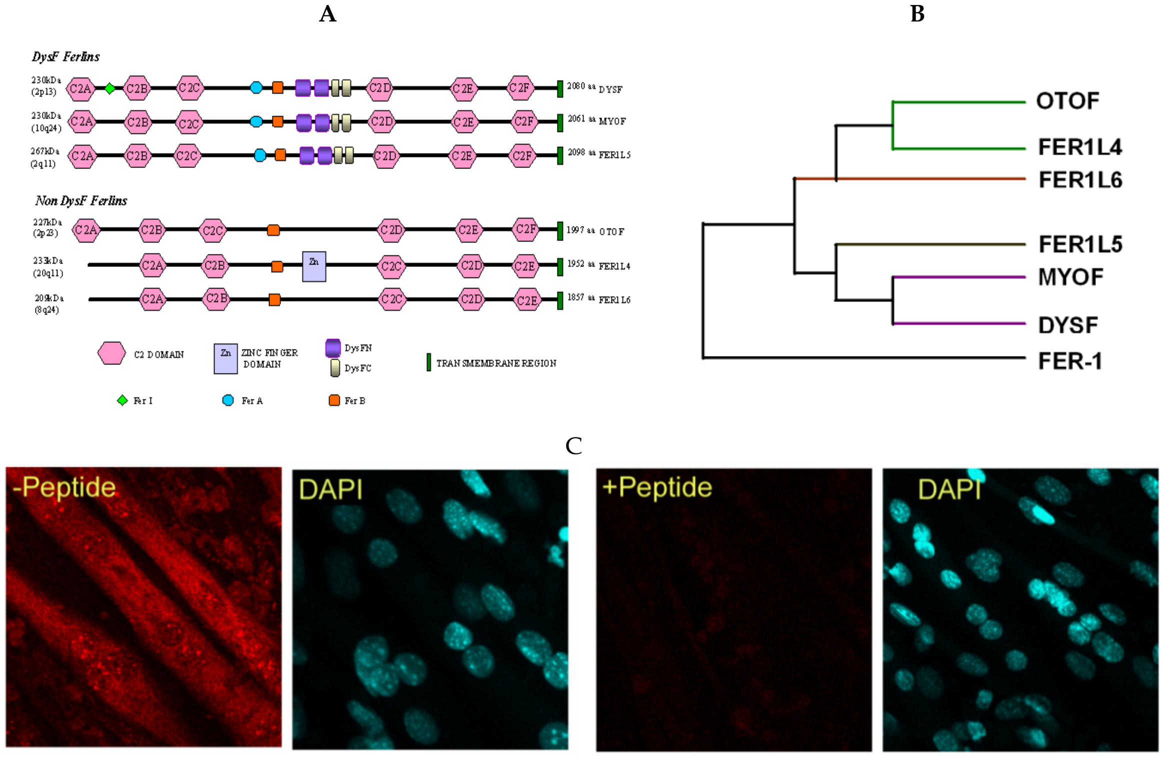

Figure S1. Schematic showing the structural similarities and subgrouping of the human ferlin protein family. (**A**) Schematic showing the structural similarities and subgrouping of the human ferlin protein family.All proteins contain tandem C2 domains,C-terminal transmembrane domain, DysFC, DysFN, Fer A, Fer B and Fer I. Fer1L4 has a zinc finger domain. (**B**) Ferlin C2 domainphylogeny.The tree is constructed based on the human ferlin C2 domains aligned using ClustalW to show the similarities of the ferlins. (**C**) Fer1L5 staining in C2C12 myotubenuclei. Sameconfocal settings wereused to capture the myotubeimages following peptide blocking as shown in Figure 1C. No specific staining was detectedin the + peptide myotube. DAPI stains nuclei. Scale bar, 15μm.

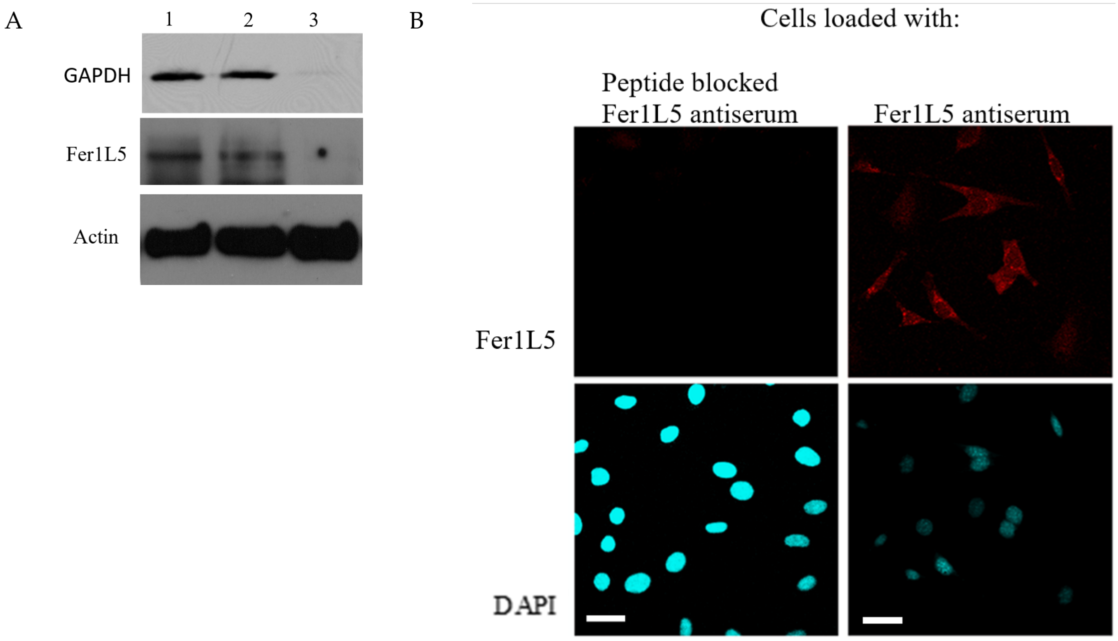

Figure S2. Transfection in the Fer1L5shRNA and Fer1L5 antibody loaded cultures. (**A**) Western blots showing 1=wild type (no transfection), 2= Mock (no target) siRNA, 3=GAPDH siRNA and Fer1L5 shRNA.The data shown is for time point 72hafter transfection. (**B**) The identical confocal setting were used to collect Z-stack images of the C2C12 cells loaded with FerIL5 anti-serum and peptide blocked FerIL5 anti-serum respectively. Scale bar, 10μM. DAPI was used to show the negative staining.

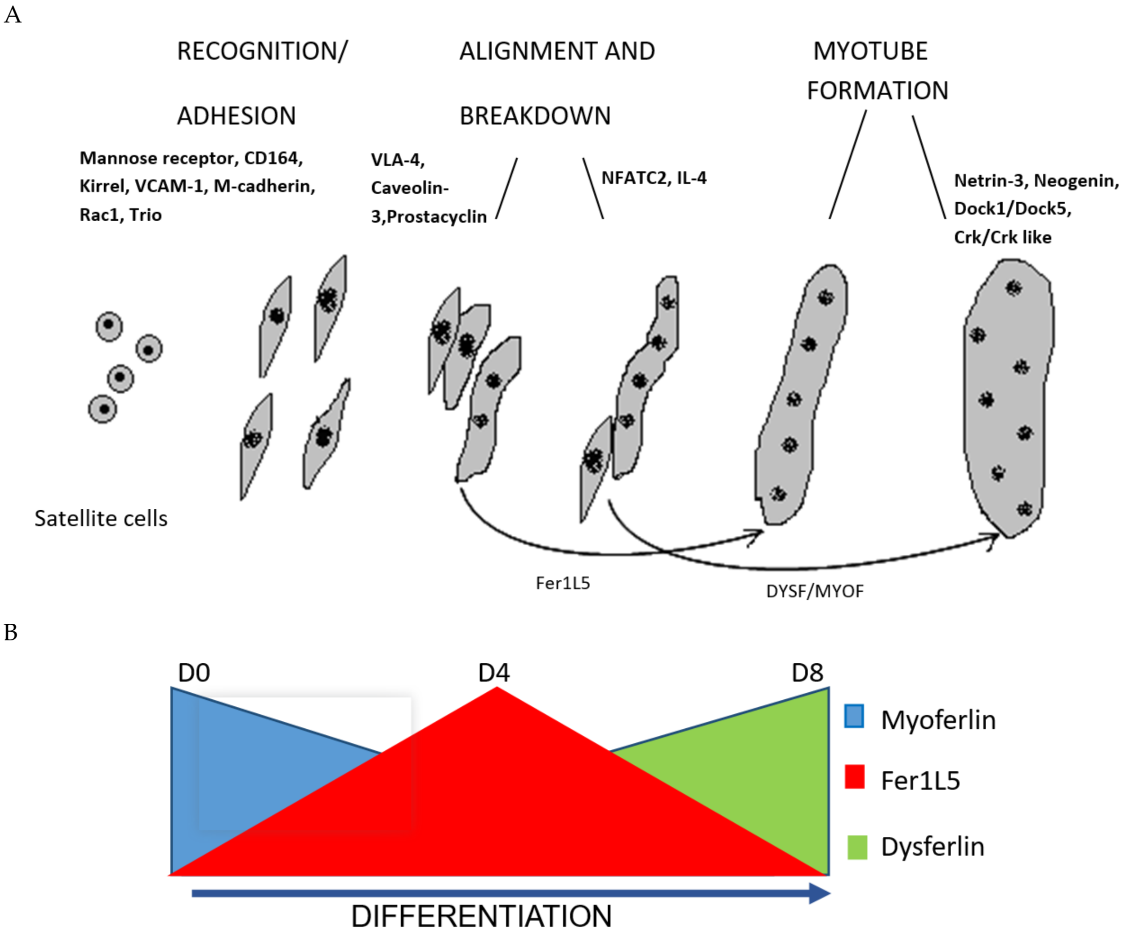

Figure S3. Fer1L5 differentiation role in myoblast (**A**) Fer1L5 role in myoblast fusion. Model showing that Fer1L5 isinvolved in the first phase and the dysferlin and myoferlin is involved in the second phase of myoblast fusion. Other proteins, which are involved in the different stages during muscle development, are shown in bold letters. (**B**) We modified the figure from Posey *et al.,*(2010) [1] to show that the Fer1L5 expression (red) is intermediate between myoferlin(skyblue) and dysferlin (light green) during C2C12 myoblastdifferentiation.

Model: The reduced formation of myotubes following Fer1L5 inhibition would be expected to disrupt the formation of prefusing myoblast/myotube and myoblast/myotube connections resulting in inefficient alignment, adhesion and fusion of the myoblast/myotube membranes (Supplementary Figure S3A). This model may potentially explain why Fer1L5 inhibition impairs the formation of large myotubes (Supplementary Figure S3B).

Reference

1. Posey C.,Lowry,PB., Roberts TL.,Ellis TS.2019.roposing the online community self-disclosure model: the case of working professionals in France and the U.K. who use online communities. *Eur J Inf Syst.* **2019**,*19*,181–195.

Publisher's Note: MDPI stays neutral with regard to jurisdictional claims in published maps and institutional affiliations.

© 2020 by the authors. Licensee MDPI: Basel, Switzerland. This article is an open access article distributed under the terms and conditions of the Creative Commons Attribution (CC BY) license (http://creativecommons.org/licenses/by/4.0/).
